# Pharmacological and non-pharmacological strategies in coronavirus disease 2019: A literature review

**DOI:** 10.1016/j.amsu.2022.103709

**Published:** 2022-05-08

**Authors:** Francisco J. González-Ruiz

**Affiliations:** Department of Cardiovascular Critical Care, National Institute of Cardiology “Dr. Ignacio Chávez”, México City, Mexico

**Keywords:** Antivirals, Monoclonal antibodies, Oxygen therapy, Mechanical ventilation, ECMO

## Abstract

The impact on mortality associated with covid-19 today exceeds five million deaths worldwide, and the number of deaths continues to rise. The complications of the survivors, socio-economic implications at a global level, economic limitations in the health systems, and physical and emotional exhaustion of health personnel are detrimental. Therapeutic strategies are required to limit the evolution of the disease, improve the prognosis of critically ill patients, and, in countries with low purchasing power, create affordable alternatives that can help contain the evolution towards the severity of infected people with mild to moderate symptoms. The misinformation and myths that today are more frequent on social networks and the implementation of practices without scientific support is a problem that aggravates the general panorama. This review aims to concentrate on the best evidence for treating SARS-CoV-2 infection in a simple and summarized manner, addressing therapies from their bases to the most innovative alternatives available today.

## Highlights

• The pathophysiological bases of classic ADRS differ significantly from those related to ARDS due to COVID-19.
• The therapeutic objective based on the pathophysiological aspects could improve the clinical evolution of the affected patients.
• The objectives set for oxygen saturation should be reconsidered since oxygen in high concentrations could have deleterious effects, especially in this patient population.
• Extracorporeal membrane circulation should not be left aside, and early implementation could save many lives in well-selected patients.

## Abbreviations

**ARDS**: acute respiratory distress syndrome
**COVID-19**: coronavirus disease
**FiO**_**2**_: fraction of inspired oxygen
**HPV**: hypoxic pulmonary vasoconstriction
**SpO 2**: oxygen saturation
**PaO**_**2**_: partial pressure of oxygen
**PEEP**: positive end-expiratory pressure
**V/Q**: ventilation-perfusion
**ACE2**: angiotensin-converting enzyme 2
**TMPRSS2**: transmembrane serine protease 2
**SARS-CoV-2**: severe acute respiratory syndrome coronavirus 2
**5-HT2**: 5-hydroxy-tryptamine
**ECMO**: Extracorporeal Membrane Oxygenation

## 1 Introduction

The severe acute respiratory syndrome coronavirus 2 pandemic has continued to impact global health. However, while immunity acquired by vaccines has been developed, 40% of the world's population has still not been vaccinated. There is no doubt that the best strategy continues to be prevented through vaccination and limiting the severity of the disease among infected people. However, despite the complete vaccination scheme, the admission of critically ill patients who require medical assistance continues. Discussions regarding treatment focused on pathophysiological aspects are fundamental. We begin the discussion of these therapeutic alternatives, highlighting some reflections regarding oxygen therapy and ventilation, which have been little noticed during the pandemic and are of great importance.

### 1.1 Oxygen therapy and mechanical ventilation

Although the goal is to maintain the oxygen saturation (SpO_2_) level between 92% and 96% in patients infected with SARS-CoV-2, we believe that this goal should be reconsidered [1]. Pathophysiological aspects of ARDS due to COVID-19 differ significantly from those of conventional ARDS. Hence, we address some aspects of this measure's importance.

Hypoxia plays a limited role in dyspnea, unlike hypercapnia, which causes dyspnea. Under conditions of mild hypoxemia (PaO_2_ 60–65), the effects on the respiratory drive are imperceptible, and dyspnea develops when PaO_2_ falls below 40 mm Hg [2]. The initial response to an acute change in this value significantly increased ventilation and respiration. Hyperpnea and tachypnea are two vital signs of hypoxemic respiratory failure [2]. Guan et al. [3] reported that only 18.7% of patients with COVID-19 had dyspnea, despite a low PaO_2_/FiO_2_ fraction value.

Additionally, constitutional factors, such as age and diabetes mellitus, dampen the response to hypoxia, especially in populations over 65 years of age [3,4]. The administration of oxygen at high concentrations (FiO2 >0.6) increases the oxygen tension in the alveoli at a low level of ventilation, which inhibits HPV [5,6].

**Fig. 1:**
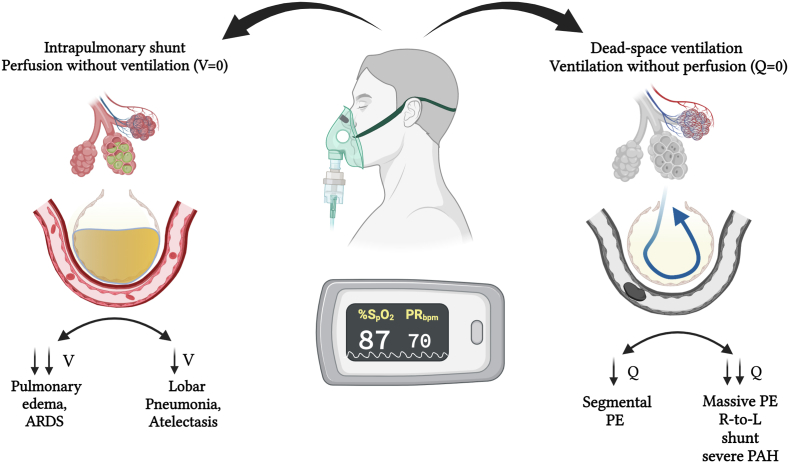
**Spectrum of ventilation/perfusión (V/Q) mismatch in COVID-19.** Multiple pathophysiological abnormalities coexist in the context of COVID-19 infection. Injury induced by direct or indirect damage leads to the release of cytokines and reactive oxygen species (ROS) and subsequently increased paracellular permeability with alveolar edema. Existing prothrombotic effects impair perfusion, leading to severe hypoxia. In neither of these scenarios does the administration of supplemental oxygen increase PaO2.

Gas exchange units with decreased or absent blood flow (high V/Q) generate increased dead space and reduced ventilation, represented by the difference in the alveolar-arterial oxygen concentration, composition of the gases in venous blood, and consequently wasted breathing. (Fig. 1)

Of the five existing causes of pulmonary hypoxemia, shunting and low V/Q mismatch substantially explain some of the pathophysiological aspects of COVID-19, and none of these respond well to supplemental oxygen [7].

In the presence of shunting (low V/Q), the increase in FiO_2_ that attempts to improve oxygenation is not particularly effective because it does not enhance the PaO_2_ of non-ventilated units, and the extra amount of oxygen in the circulation cannot compensate for the impact of the change in blood flow [8,9]. In this context, it is essential to mention that the administration of high oxygen concentrations can be associated with reabsorption atelectasis, increasing nitrogen removal and allowing the diffusion of inspired oxygen into the blood, causing alveolar collapse [10,11].

**Fig. 2:**
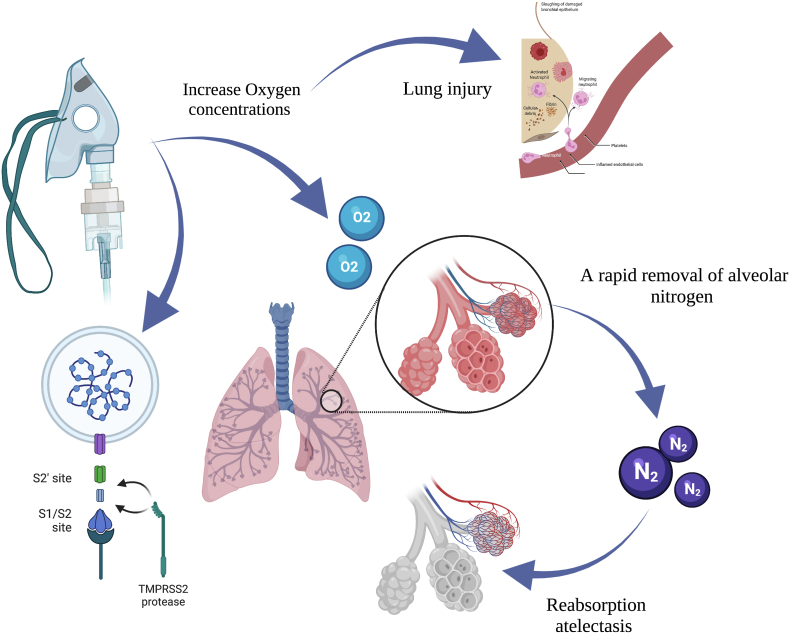
**Effects of high concentrations of oxygen.** The toxic effects of oxygen have been widely described. Lung injury induced by free radical generation resembles injury in respiratory distress syndrome. High concentrations of oxygen increase airway resistance, which is reversible in the short term but becomes refractory sometime later. Moreover, changes related to lobar hepatization and formation of hyaline membranes are shown with proliferative pneumonitis that affects alveolar epithelium and fibroblasts. It should not be forgotten that high concentrations produce reabsorption atelectasis; Increased oxygen concentrations cause alveolar nitrogen to be rapidly removed, increasing oxygen diffusion into the bloodstream causing alveolar collapse.

Myti et al. [11] demonstrated that an increase in FiO_2_ concentration at high doses increased the expression of receptors and co-receptors for the entry of SARS-CoV-2, including ACE2. The expression of transmembrane serine proteases TMPRSS1, TMPRSS, and TMPRS11D in airways, lungs, and intestinal epithelial cells was four times higher in the first 48 h of oxygen administration, with an average FiO_2_ of 0.85 and was related to severe deterioration of affected patients [11]. (Fig. 2)

Interventions that could improve hypoxemia in shunts include increasing mixed venous oxygen saturation, increasing hemoglobin concentration, decreasing oxygen consumption, and increasing cardiac output. Paradoxically, it has been shown that the shunt fraction worsens significantly with increased cardiac output and may be less effective than planned [12,13].

Multiple investigations have demonstrated that the administration of non-invasive positive pressure ventilation increases the total dead space due to increased airway pressure, which results in compression of alveolar capillaries in non-dependent pulmonary regions [14,15].

Furthermore, the association between higher positive end-expiratory pressure (PEEP) (lower PaO_2_/FiO_2_), worsening hypoxemia, and a higher fraction of physiological dead space (higher ventilatory ratio) point to vascular dysregulation disproportionately aggravated by redirection of perfusion away from overdistended oxygenated alveoli, without the benefits of additional lung recruitment, consistent with recent studies on the variable efficacy of lung recruitment in COVID-19-induced ARDS [14,15].

Finally, the effects of PEEP on dead space could be attenuated if the negative impact of PEEP on cardiac output was counteracted by fluid loading to increase cardiac preload, which also indicates that the reduction of cardiac output could significantly increase the dead space [[14], [15], [16]]. Despite capillary leakage, existing pulmonary interstitial edema can worsen, further deepening the deterioration of gas exchange. In all cases requiring mechanical ventilation due to the measures refractoriness, the intervention should not be delayed [[15], [16], [17], [18]]. In mechanically ventilated patients, the use of neuromuscular blockers for 48 h with continuous infusion additionally demonstrated a benefit in terms of mortality at 90 days. These results were linked to cisatracurium in the ACURASYS trial [19,20].

The prone position decreases shunt and V/Q mismatch, improves oxygenation, and significantly impacts survival. This intervention is not related to an improvement in gas exchange but rather to a more uniform distribution of regional ventilation and eversion of the vascular redistribution that leads to minor lung injury and should be implemented in the admission of patients with hypoxemia [20]. Lung recruitment and perfusion from this therapy improved the 90-day mortality in the PROSEVA trial. The prone position has also been shown to relieve right ventricular tension secondary to increased pulmonary vascular resistance in the context of hypoxemia and hypercapnia [21].

### 1.2 Glucocorticoids

The use of glucocorticoids in the early stages of the pandemic has been a controversial issue. Although they are used in other types of severe viral pneumonia (SARS, seasonal, and avian influenza) [[22], [23], [24]], some contraindications in immunosuppressed patients and the risk of bacterial superinfections have been a concern.

The first small-scale trial in China showed promising results with improved clinical outcomes using methylprednisolone [25]. It was not until the results of the RECOVERY trial that its use was standardized on a large scale [26]. Of the primary outcomes, the results showed a significant decrease in mortality at 28 days, reduced need for mechanical ventilation, clinical improvement in patients treated conventionally during mechanical ventilation, and those who received oxygen therapy [26]. The benefits of this therapy did not extend to patients who did not require respiratory support [26]. Among the secondary outcomes, the hospitalization rate decreased considerably in the dexamethasone group, and the 28-day discharge goal was higher for patients who received ventilatory assistance.

Additionally, steroids could limit relative adrenal insufficiency in patients with COVID-19. A recent study published by Perez-Torres et al. [27] demonstrated that the presence of immunoglobulin E anti-adrenocorticotropic hormone antibodies with decreased plasma cortisol levels, despite the administration of steroids as part of the treatment protocol, attenuated the response of the hypothalamic-pituitary-adrenal axis, which could play an essential role in the pathophysiology of severe disease due to COVID-19 [27].

### 1.3 Nucleosides analogs

The prototype drug remdesivir, used in other lethal infectious entities (Ebola and Nipah viruses), has shown practical antiviral effects [[28], [29], [30]]. It induces the inhibition of coronavirus RNA polymerase replication in respiratory epithelial cells, leading to a decrease in the viral load in lung cells [31,32]. The use of remdesivir for ten days reported by Beigel et al. [32] showed a reduction in the number of days for recovery and hospitalization, with no impact on overall mortality.

### 1.4 Monoclonal antibodies

#### 1.4.1 Tocilizumab

It is a monoclonal antibody directed against the IL-6 receptor used in inflammatory rheumatological diseases [33,34]. The use of tocilizumab in endothelial dysfunction and increased vascular permeability due to COVID-19 showed benefits in the resolution of symptoms, reduced oxygen use, and the need for mechanical ventilation [[35], [36], [37]]. However, more recently, the results of the COVACTA trial [38], which were later confirmed by the REMDACTA trial [39], were disappointing, as they did not show any benefit in mortality or length of hospitalization with the use of this drug.

#### 1.4.2 Sotrovimab

The second monoclonal antibody acts on the spike protein (S), preventing the adherence and penetration of the virus into host cells [40]. It is a monoclonal antibody designed for patients over 12 years of age and over 40 kg with positive test results for SARS-CoV-2, initial outpatient symptoms, and no supplemental oxygen. These results are promising, although premature [40,41]. The administration of these drugs to hospitalized and critically ill patients is associated with worse clinical outcomes [41].

#### 1.4.3 REGEN-COV (Ronapreve)

This combination of monoclonal antibodies (casivirimab and imdevimab) acts by binding to the epitopes of the binding domain in the peak protein of SARS-CoV-2, maintaining efficacy in the neutralization of variants of concern and apparently with effectiveness against variant resistance. The trial results showed a significant reduction in viral load and faster recovery rates than those in the control group. In the sub-analysis of the study, this combination of antibodies was administered preventively to minors between 12 and 17 years of age, showing that none of the infected patients had symptoms related to the infection. In the same sub-analysis, only 2% of participants under 50 years of age showed symptomatic disease [42].

Following the immunomodulatory pathway, two potent monoclonal antibodies initially discovered in convalescent plasma from a COVID-19 patient and later developed by Eli Lilly were bamlanivimab and etesevimab [43]. The results of phase 2 and those published at the beginning of phase 3 of the BLAZE-1 trial showed bamlanivimab as monotherapy, and the combined use of bamlanivimab and etesevimab significantly reduced hospitalization and progression of the clinical condition [43,44]. Therefore, they received authorization from the Food and Drug Administration (FDA) for emergency use [45,46]. Candidate patients for therapy (mono- or combined therapy) were outpatients over 12 years of age with mild-to-moderate disease, with a significant risk of progression to severe illness. Final results of phase 3 of the BLAZE-1 trial [47] showed a reduction in mortality associated with the use of these drugs, in addition to a 16-fold reduction in viral load when bamlanivimab and etesevimab were used together, compared with the control group [47]. Although low (estimated at 1.4% for bamlanivimab and 13.3% for bamlanivimab and etesevimab), the adverse effects were considerable, including nausea, rash, dizziness, diarrhea, and systemic arterial hypertension.

The use of a combination of monoclonal antibodies (tixagevimab and cligavimab) (Evusheld) was recently approved by the FDA for pre-exposure use [48]. As previously mentioned, these antibodies have an affinity for different sites of the SARS-CoV-2 peak protein, showing reduced Fc receptor and C1q complement binding. Phase III results in the PROVENT assay show prolonged action of the drug, which could confer protection for up to 12 months in a single application [49,50]. They were designed to be administered to patients older than 12 years who did not show adequate immunity after vaccination or in cases with contraindications. The effects of medication in general use will still have to be detailed on a large scale. An additional concern is a cost of acquiring these medications if they become a part of current innovative drugs that could limit the effects of the pandemic.

### 1.5 Janus kinase inhibitor

Following inflammatory dysregulation and attempts to mitigate it, analysis of algorithms using artificial intelligence suggested baricitinib. Janus kinase 1 and 2 inhibitors act by inhibiting intracellular cytokine signaling (IL-2, IL-6, IL-10, IF-γ, and granulocyte colony-stimulating factor) and directly against the virus through the deterioration of protein kinase 1, prevention of viral entry, and decrease in cellular effectiveness. Administered at a rate of 4 mg/day or 2 mg/day for 14 days in case of impaired renal clearance (<60 ml/min), it showed significantly faster recovery rates compared to the control groups [51]. Later, the results of the Adaptive COVID-19 Treatment Trial 2 trial published by Kalil et al. [52] showed the substantial benefits of the combination of baricitinib and remdesivir in short-term recovery rates, with an improvement in the blockage of the immune cascade and a significant reduction in viral replication.

### 1.6 Novel antivirals

Efforts to find an ideal drug for fighting COVID-19 have been meritorious but have limited efficacy. Economic disparities in the acquisition of these, precariousness (such as in the case of remdesivir), and limitations in distribution have been the main limitations. The scientific and medical community has recently been interested in two antivirals: molnupiravir and paxlovid.

Molnupiravir is an antiviral agent that induces mutagenesis by introducing errors into the viral genome, targeting RNA-dependent RNA polymerase (such as nucleoside analogs) [53]. The intermediate results of the MOVe-OUT trial showed a 50% reduction in the hospitalization rate in patients who received the drug (7.3% of the patients who received molnupiravir compared with 14.1% in the control group) without having reported deaths in the group. Preliminary results have been encouraging, and the ease of administration (compared to remdesivir) makes it an ideal drug for outpatient use [53].

In contrast, preliminary phase II-III results regarding Paxlovid [54] found an 89% reduction in the hospitalization rate. It decreased mortality when used within the first three days of the onset of symptoms. Both drugs have received approval for emergency use in the United Kingdom. Although the results are preliminary, they are still encouraging. The final phase results are needed; however, it could become the drug of choice for early SARS-CoV-2 infection.

## 2 Repurposing drugs in COVID-19

As we delve deeper into the pathophysiological aspects of SARS-CoV-2 infection and consider the inequity in acquiring high-cost drugs, reconsidering the reuse of previously studied drugs appears to be an effective strategy.

Kuindersma et al. [55] proposed using serotonin receptor antagonists during the early stages of infection. In non-hypoxic conditions, serotonin appears to have implications for NO generation through the 5-HT2b receptor, which consequently causes vasodilation. In an environment of hypoxia, vasoconstriction, secondary to smooth muscle contraction and deterioration of the HPV mechanism through 5-HT2a receptors, occurs without the possibility of NO regeneration. In this context, the increase in serotonin secretion would perpetuate microvascular vasoconstriction, significantly impairing V/Q mismatch and hypoxia. As a 5-HT2a receptor antagonist, Ketanserin could reverse microvascular vasoconstriction, improving the perfusion of relatively preserved areas and, secondarily, inhibiting platelet aggregation and preventing additional serotonin formation and thrombosis [55].

Following the line of repurposing and selective serotonin reuptake inhibitor drugs, considering Fluvoxamine [56] among the repurposed drugs is inevitable. The mechanisms of action of fluvoxamine in COVID-19 are uncertain, although the anti-inflammatory and antiviral mechanisms remain unclear. In the TOGETHER trial, a randomized controlled trial, fluvoxamine 100 mg twice daily for ten days showed a clinically significant absolute risk reduction of 5% and a relative risk reduction of 32% in the hospitalization rate within the initial stage of infection. Modulation of the release of inflammatory cytokines through the activation of the S1R receptor and its antiplatelet activity resembles the effects of ketanserin [56].

This strategy for reusing low-cost, highly safe, easy-to-use, readily available, and tolerable drugs could be successful in countries with difficulties in acquiring novel drugs. Large-scale trials are necessary to consider them as practical and valuable alternatives.

### 2.1 Extracorporeal membrane oxygenation (ECMO)

After comprehensively addressing the pathophysiological aspects of severe SARS-CoV-2 infection, it is clear that the only helpful strategy in the context of shunts and V/Q mismatch is ECMO.

Preliminary reports at the beginning of the pandemic showed unfavorable results regarding the use of ECMO in ARDS secondary to COVID-19 [57]. As the pandemic evolved, mortality rates have been equated in various published cohorts, comparable to those of the extracorporeal membrane oxygenation for severe acute respiratory distress syndrome trial (35.7% in the venovenous context) [58]. The diversity in the results may be due to the heterogeneity of the criteria for including patients in therapy and should be considered using a multidisciplinary group, evaluating the benefit to the patients, the impact of additional patients, and the health systems to which they belong. Favorable results have been demonstrated in patients who underwent ECMO three days after initiating mechanical ventilation [59].

**Fig. 3:**
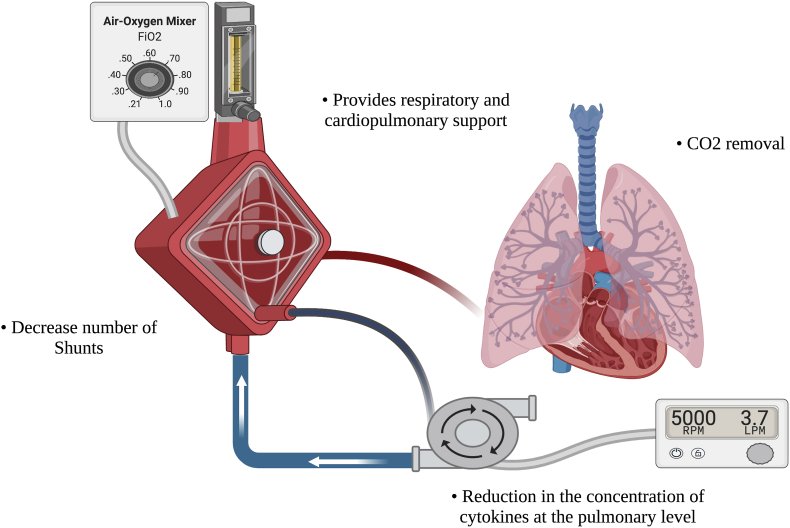
**Benefits of extracorporeal membrane circulation (ECMO).** The benefits of this intervention extend to respiratory and cardiovascular support, effectively removing concentrations of carbon dioxide (CO2), significantly reducing concentrations of pulmonary inflammatory cytokines, and eliminating shunts.

The selection criteria for the initiation of ECMO therapy were as follows: a P/F ratio <80 mmHg for more than 6 h; P/F radius <50 mmHg for more than 3 h; pH < 7.25 with an increase in the partial pressure of CO_2_ of >60 mmHg, which is maintained for more than 6 h [56,57]. Poor prognostic factors associated with ECMO therapy include age, chronic lung disease, renal failure before treatment, immunocompromised status, low body mass index, and cardiac arrest before the intervention [59]. Complications associated with ECMO therapy include medical and intracerebral hemorrhage prevalent in up to 12% of patients, including those related to the circuit (circuit change, membrane failure, cannulation problems, and cannulae) and pump failure [[59], [60], [61]]. (Fig. 3)

Although controversy persists, ECMO therapy should be considered in the care protocols for critically ill patients with ARDS secondary to COVID-19. The refractoriness of initial treatment, improvement in the patient selection protocols, and early use of the device should encourage multidisciplinary teams not to rule out therapy as long as it is within reach of the care services [[62], [63], [64], [65]].

## 3 Conclusion

The evidence gathered throughout the COVID-19 pandemic strengthens the bases related to a disproportionate inflammatory state impacting the vascular endothelium, leading to catastrophic consequences. Disparities in symptoms and severity are not yet fully defined, but genetic factors and diversity in viral entry pathways offer explanations. The dominant variants worldwide are delta and omicron, with overwhelming dominance. The increased potential for omicron transmissibility and its non-serious symptoms could have led to the end of the pandemic. However, severe delta variant cases and the unvaccinated population who do not have the means to get vaccinated or refuse to adopt this preventive strategy will continue to need hospitalization in intensive care units. We trust that this document can contribute to the unification of care efforts and generate great success in the critical care of patients with COVID-19.

## Human subjects/informed consent statement

Not applicable.

## Data availability statement

Not applicable.

## Provenance and peer review

Not commissioned, externally peer reviewed.

## Ethical approval

Not applicable.

## Sources of funding

No funding.

## Author contribution

This manuscript was written entirely by this author.

## Registration of research studies

1 Name of the registry: Not applicable
2 Unique Identifying number or registration ID: Not applicable
3 Hyperlink to your specific registration (must be publicly accessible and will be checked): None

## Guarantor

I take full responsibility for the publication of the manuscript:

Dr. Francisco Javier González Ruiz.

## Declaration of competing interest

The author declares that there is no conflict of interest.
